# Imaging Biomarkers to Predict and Evaluate the Effectiveness of Immunotherapy in Advanced Non-Small-Cell Lung Cancer

**DOI:** 10.3389/fonc.2021.657615

**Published:** 2021-03-19

**Authors:** Ying Liu, Minghao Wu, Yuwei Zhang, Yahong Luo, Shuai He, Yina Wang, Feng Chen, Yulin Liu, Qian Yang, Yanying Li, Hong Wei, Hong Zhang, Chenwang Jin, Nian Lu, Wanhu Li, Sicong Wang, Yan Guo, Zhaoxiang Ye

**Affiliations:** ^1^ Department of Radiology, Tianjin Medical University Cancer Institute and Hospital, National Clinical Research Center for Cancer, Key Laboratory of Cancer Prevention and Therapy, Tianjin, Tianjin’s Clinical Research Center for Cancer, Tianjin, China; ^2^ Department of Medical Imaging, Cancer Hospital of China Medical University, Liaoning Cancer Hospital and Institute, Shenyang, China; ^3^ Department of Medical Oncology, 1st Affiliated Hospital, Zhejiang University School of Medicine, Hangzhou, China; ^4^ Department of Radiology, 1st Affiliated Hospital, Zhejiang University School of Medicine, Hangzhou, China; ^5^ Department of Radiology, Hubei Cancer Hospital, Tongji Medical College, Huazhong University of Science and Technology, Wuhan, China; ^6^ Department of Thoracic Oncology, Cancer Center, West China Hospital, Sichuan University, Chengdu, China; ^7^ Department of Radiology, West China Hospital, Sichuan University, Chengdu, China; ^8^ Department of Radiology, Tianjin Chest Hospital, Tianjin, China; ^9^ Department of Radiology, The First Affiliated Hospital of Xi’an Jiaotong University, Xi’an, China; ^10^ Department of Radiology, Sun Yat-sen University Cancer Center, State Key Laboratory of Oncology in Southern China, Guangzhou, China; ^11^ Department of Medical Imaging, Shandong Cancer Hospital and Institute, Shandong First Medical University and Shandong Academy of Medical Sciences, Jinan, China; ^12^ Prognostic Diagnosis, GE Healthcare China, Beijing, China

**Keywords:** immunotherapy, non-small-cell lung cancer, imaging biomarkers, response prediction, radiomics, Delta-radiomics

## Abstract

**Objective:**

We aimed to identify imaging biomarkers to assess predictive capacity of radiomics nomogram regarding treatment response status (responder/non-responder) in patients with advanced NSCLC undergoing anti-PD1 immunotherapy.

**Methods:**

197 eligible patients with histologically confirmed NSCLC were retrospectively enrolled from nine hospitals. We carried out a radiomics characterization from target lesions (TL) approach and largest target lesion (LL) approach on baseline and first follow-up (TP1) CT imaging data. Delta-radiomics feature was calculated as the relative net change in radiomics feature between baseline and TP1. Minimum Redundancy Maximum Relevance (mRMR) and Least Absolute Shrinkage and Selection Operator (LASSO) logistic regression were applied for feature selection and radiomics signature construction.

**Results:**

Radiomics signature at baseline did not show significant predictive value regarding response status for LL approach (*P* = 0.10), nor in terms of TL approach (*P* = 0.27). A combined Delta-radiomics nomogram incorporating Delta-radiomics signature with clinical factor of distant metastasis for target lesions had satisfactory performance in distinguishing responders from non-responders with AUCs of 0.83 (95% CI: 0.75–0.91) and 0.81 (95% CI: 0.68–0.95) in the training and test sets respectively, which was comparable with that from LL approach (*P* = 0.92, *P* = 0.97). Among a subset of those patients with available pretreatment PD-L1 expression status (n = 66), models that incorporating Delta-radiomics features showed superior predictive accuracy than that of PD-L1 expression status alone (*P <*0.001).

**Conclusion:**

Early response assessment using combined Delta-radiomics nomograms have potential advantages to identify patients that were more likely to benefit from immunotherapy, and help oncologists modify treatments tailored individually to each patient under therapy.

## Introduction

In recent years, immunotherapies have provided durable clinical responses and demonstrated a survival benefit across a variety of cancer types, including non-small cell lung cancer (NSCLC) (1–5). Immune-checkpoint inhibitors (ICIs) targeting programmed death 1 (PD-1) or its ligand programmed death ligand 1 (PD-L1) are recommended by the National Comprehensive Cancer Network (NCCN) (6) and the European Society of Medical Oncology (ESMO) (7) for locally advanced and metastatic NSCLC without targetable genetic alterations. Despite their remarkable success, increased progression-free survival (PFS) and/or overall survival (OS) remains limited to only a small proportion (15–30%) of patients according to published evidence (8–10). There is therefore a need for the development of methods to identify patients who are most likely to respond to immunotherapy.

Several biomarkers which are currently used for the selection of patients eligible for cancer immunotherapy, such as PD-L1 expression and tumor mutation burden (TMB), have achieved clinical relevance to some extent (11, 12). However, there are many challenges concerning the effective use of them as predictive biomarkers, including inadequate sample tissue for reliable PD-L1 quantification and whole-exome sequencing (WES), heterogeneous expression due to intra-tumoral heterogeneity (13), absence of standardization between different tests (14), and increasement of diagnostic complexity and cost. Another issue is that several studies revealed that patients with PD-L1 negative tumors could still derive clinical benefit from ICIs (15–17). Thus, the insufficiency of current biomarkers highlights the urgent need to identify novel predictive biomarkers for a better stratification of patients receiving ICIs.

Radiomics, an emerging field within medical imaging, is capable of generating imaging biomarkers as decision support tools for clinical practice (18). Under the motivation that biomedical images contain information that reflects underlying pathophysiology, recent studies have proposed radiomics approach to predict response to ICIs (19–24). Nevertheless, further evaluation needs to be carried out in translating such research into clinical practice because most literature in the field had a multi-localization/multi-type tumor cohort design. Delta-radiomics features (Delta-RFs) which capture therapy-induced changes in radiomics features are now being evaluated as a complement to Response Evaluation Criteria in Solid Tumor (RECIST) criteria for monitoring therapeutic response in several tumor types (25–31). Khorrami et al. showed preliminary evidence for clinical use of Delta-radiomics calculated from contrast-enhanced CT images as predictive biomarkers of response to ICIs therapy in NSCLC (31). However, contrast can obscure radiomics textural features (32), and the heterogeneity of contrast-enhanced protocols across institutes magnifies the concern about reproducibility of radiomics. In the current study, we aim to develop and validate radiomics/Delta-radiomics nomograms incorporating clinical factors and plain CT imaging data to predict response to ICIs in patients with advanced NSCLC. Also, we compared the predictive efficacy of Delta-radiomics models against pretreatment PD-L1 expression status.

## Materials and Methods

### Study Design

**Figure 1:**
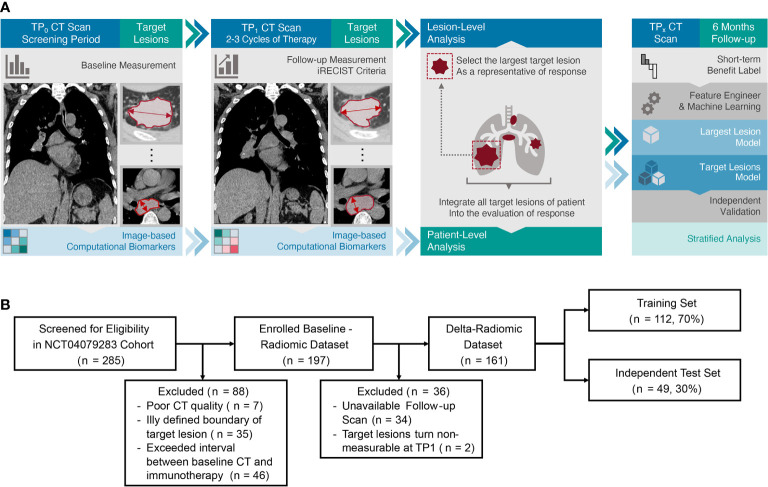
**(A)** Study workflow. The workflow presented a summary of target lesions annotation and response assessment, preprocessing and modeling schemes of radiomics. **(B)** Patient flow diagram. For baseline-radiomic dataset, training and test set were randomly divided in a proportion of 7:3 respectively as well.

This retrospective multicenter study was conducted in accordance with the Declaration of Helsinki and was approved by ethics committee of each participating hospital, with the requirement for informed consent waived. Between August 1, 2016 and February 28, 2019, radiologic image archives of nine participating institutions were searched consecutively to identify patients. The inclusion criteria were as follows: (a) histologically confirmed NSCLC; (b) immunotherapy with PD-1 ICIs at first or later line; (c) available baseline demographics and CT images prior to therapy; (d) follow-up time from initiation of immunotherapy was at least 6 months with regular clinical evaluations and CT scans after each two or three cycles of ICIs. The exclusion criteria were (a) CT images were of poor quality; (b) the boundary of target lesion was ill defined on plain CT scan and contrast-enhanced CT images were not available as reference; (c) time between baseline imaging and immunotherapy treatment exceeded four weeks. Finally, 197 patients were enrolled for baseline analysis, then the entire cohort was randomly divided into a training set (n = 137) and an independent test set (n = 60) at a ratio of 7:3. The same procedure was applied to a sub-group of patients (n = 161) who had available CT scans at baseline (time point 0, TP0) and the end of the second cycle of immunotherapy (time point 1, TP1), and this sub-group was used to perform a time-dependent analysis (**Figure 1**). Clinical characteristics (age at diagnosis, gender, smoking history, pathological type, and TNM stage) of all patients were obtained from the medical records.

### Imaging Data Acquisition and Harmonizing

The pretreatment and follow-up CT scans were acquired on a varied set of CT scanners (**Supplementary Data**). The median time interval between baseline CT examination and initiation of immunotherapy was 12 days. For preprocessing, all CT images were resampled to 1.5 mm resolution on all three directions to standardize the voxel size across patients. In addition, z-score normalization was applied to unify CT-value scales across scanners.

### Tumor Delineation and Treatment Response Assessment

Two radiologists (YL, with 13 years of experience in thoracic radiology and MW, with 3 years of experience in thoracic radiology) who were blinded to the outcome label reviewed baseline CT images and defined the target lesions according to RECIST 1.1 (33) (maximum of five lesions, two per organ) in consensus, and then the largest target lesion was chosen for each case. Totally, 322 target lesions were identified for all patients. Then the volume of interest (VOI) of all target lesions on plain CT images (both baseline and follow-up scans) were delineated manually *via* ITK-SNAP (www.itksnap.org) by one radiologist (MW) and then reviewed and modified by another radiologist (YL).

We classified response patterns on a patient basis. Clinically, immunotherapy response is frequently measured at 6 months (19, 34). Therefore, the endpoint of our study was a dichotomous response status (responder/non-responder), as defined by iRECIST (35) at 6 months of immunotherapy initiated, which was convinced that had better representative of benefits. Patients presenting complete response (CR/iCR), partial response (PR/iPR) or stable disease (SD/iSD) were considered as “responders”, patients who had confirmed progressive disease (iCPD) after treatment were classified as “non-responders”. For those patients who were thought to be unconfirmed progression (iUPD) at 6-month follow-up, their response status was determined by additional follow-ups to ensure unconfirmed progression would not be used as labels in model training.

### Detection of PD-L1 Expression Status

PD-L1 expression was measured through IHC testing with biopsy or resection specimens, and a minimum of 100 tumor cells (TCs) were required for the assessment. PD-L1 expression was quantified by the tumor proportion score (TPS), which is defined as percentage of PD-L1-positive TCs over total TCs, and it was classified into two levels: negative expression (TPS <1%), and positive expression (TPS ≥1%) owing to the diversity of pathological reports in our dataset.

### Feature Engineering and Signature Building

About 402 handcraft radiomics features (RFs) were extracted using in-house software (Analysis Kit, version 3.2.5, GE Healthcare) (**Table S1**). For patients who received baseline and follow-up CT scan at TP1 (median: 52 days), RFs were extracted from both time points respectively. The Delta-RFs, which were transmitted into the same analysis workflow as baseline RFs, were defined as the relative net change of RFs between TP0 and TP1 (Equation (1)):

$$\text{Relative Net Change}=\left(\text{Feature}{\;}_{\text{TP}1}-\text{Feature}{\;}_{\text{TP}0}\right)/\text{Feature}{\;}_{\text{TP}0}$$

To choose the optimal subset of features, Minimum Redundancy Maximum Relevance (mRMR) was performed to eliminate redundant and irrelevant features in advance. Then the Least Absolute Shrinkage and Selection Operator (LASSO) logistic regression was conducted to construct the final model. A radiomics signature (Radscore) was calculated for each patient *via* a linear combination of selected features and coefficient vector. Besides, two approaches of organizing Radscore or Delta-Radscore were proposed to promote lesion-wise analysis toward individual-wise on the assumption that lesion-wise response might not act as a global representative of patient benefit from immunotherapy due to those complicated individual response patterns.

Largest target lesion (LL) approach: select RFs or Delta-RFs of the largest target lesion as individual-wise signature to predict therapy response.

Target lesions (TL) approach: in single-time-point analysis, average RF of all target lesions is regarded as a global image biomarker passed to further analysis, whereas in Delta-radiomics analysis, relative net change of average RF is used instead.

### Statistical Analysis

All statistical analyses were performed using R (version 3.5.1) and Python (version 3.5.6). Chi square test was used for categorical variables. Independent *t*-test or Mann–Whitney test was used for continuous variables. A multivariate logistic regression analysis with backward elimination method was performed to construct the best model combining clinical factors and RFs. Performance of the models were evaluated with area under the ROC curve (AUC). Differences between various AUCs were compared with the DeLong test (36). Calibration curves were applied to evaluate the predictive accuracy of the nomogram model generated. To evaluate clinical utility of the radiomics nomogram, decision curve analysis (DCA) was performed by quantifying the net benefits at different threshold probabilities. A two-tailed *P*-value <0.05 indicated statistical significance.

## Results

### Clinical Characteristics

**Figure 2:**
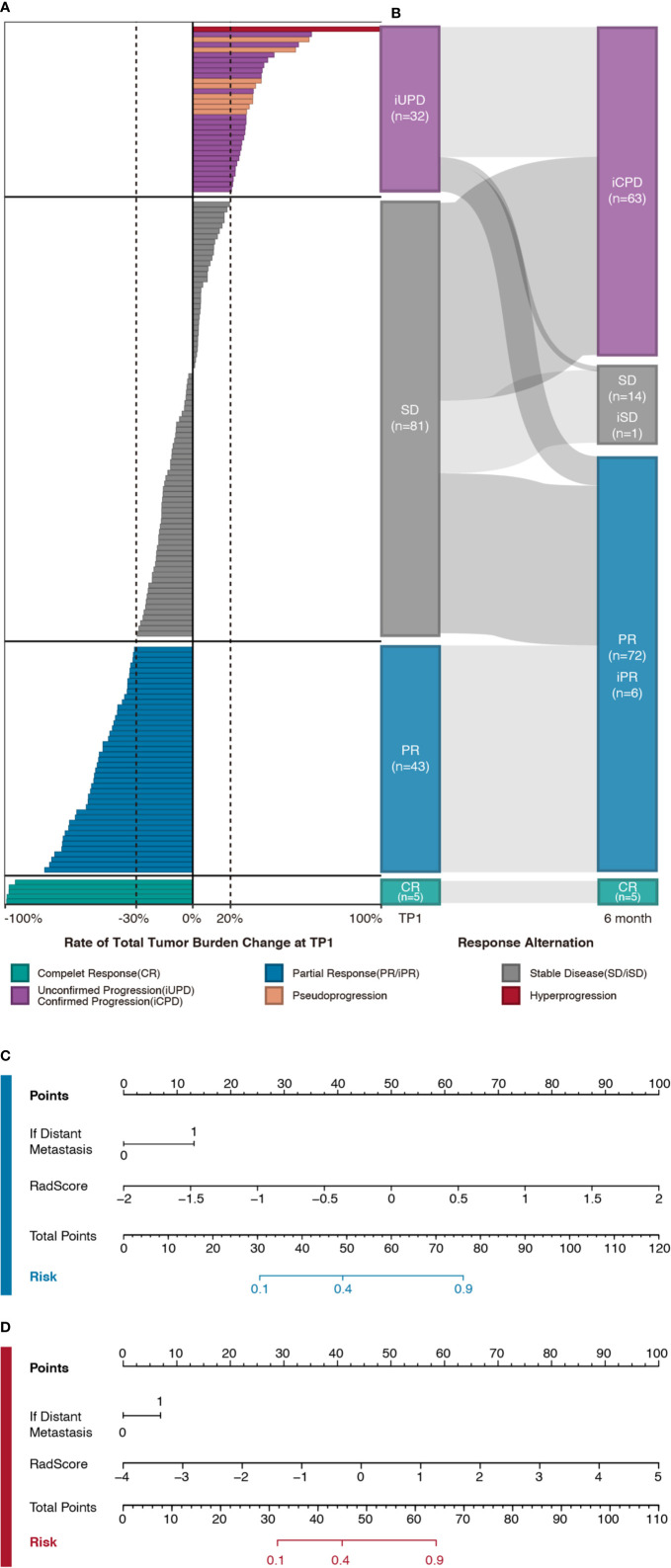
**(A)** Individual response map of patients in Delta-radiomics sub-cohort. Bars indicate the changes of total tumor burden between baseline and TP1 CT scans. Patients are grouped on the basis of therapy response at TP1 following iRECIST criteria (Complete response [CR] in green, partial response [PR] in blue, stable disease [SD] in gray, and unconfirmed progression [iUPD] in purple). In addition, hyper-progression (*n* = 1, in red) and pseudo-progression (*n* = 8, in orange) are noted as well. **(B)** Sankey diagram depicts therapy response alternation flow within follow-up interval. For those patients who met the progression threshold (20% increasement of tumor burden) at any time point within follow-up interval, updated response labels are attached according to their subsequent assessment (Confirmed progression [iCPD], stable disease [iSD], and partial response [iPR]). It’s noteworthy that for those patients who were thought to be iUPD at 6-month, their labels were determined by additional follow-ups so that any unconfirmed progression would not be used as labels in model training. **(C, D)** Nomograms of largest target lesion model (in blue) and target lesions model (in red) which were developed in training set respectively.

A total of 197 eligible patients who met the criteria were identified from nine participating hospitals. 105 patients received monotherapy with PD-1 ICIs (Nivolumab, Pembrolizumab, Tislelizumab, Sintilimab, or Camrelizumab), and 92 patients were treated with immunotherapy-based combinations (PD-1 ICIs with chemotherapy and/or antiangiogenic agents). We observed that 41.62% patients (n = 82) showed PD, and the reaming of them present PR (n = 94) or SD (n = 21) at the sixth month, with an overall disease control rate (DCR) of 58.37% (**Figures 2A, B**). There were no significant differences in DCR and clinical characteristics between the two cohorts, which justified their use as training and test sets (**Table S2**). The differences in clinical characteristics at baseline between responders and non-responders were not significant, except for distant metastasis in training set (*P* = 0.01) (**Table 1**).

**Table 1:** Characteristics of patients in baseline analysis.

Characteristics	Training set		*P* value	Test set		*P* value
	Responders	Non-responders		Responders	Non-responders	
Age, median (range)	63 (35–84)	64 (36–78)	0.52	63 (29–75)	62 (41–77)	0.86
Male	64 (36–84)	64 (36–78)		63 (29–75)	58 (41–74)	
Female	55 (43–79)	61 (37–72)		64 (43–72)	74 (62–77)	
Sex, No. (%)						
Male	68 (83.95%)	44 (78.57%)	0.42	31 (91.18%)	22 (84.62%)	0.71
Female	13 (16.05%)	12 (21.43%)		3 (8.82%)	4 (15.38%)	
Smoking history, No. (%)						
Non-smokers	22 (27.16%)	17 (30.36%)	0.68	8 (23.53%)	8 (30.77%)	0.53
Smokers	59 (72.84%)	39 (69.64%)		26 (76.47%)	18 (69.23%)	
Pathological type, No. (%)						
Adenocarcinoma	37 (45.68%)	29 (51.79%)	0.75	18 (52.94%)	13 (50.0%)	0.87
Others	44 (54.32%)	27 (48.21%)		16 (47.06%)	13 (50.0%)	
Distant metastasis, No. (%)						
Absence	21 (25.93%)	5 (8.93%)	0.01^*^	8 (23.53%)	2 (7.69%)	0.20
Presence	60 (74.07%)	51 (91.07%)		26 (76.47%)	24 (92.31%)	
Treatment strategy, No. (%)						
Monotherapy	37 (45.68%)	31 (55.36%)	0.27	18 (52.94%)	19 (73.08%)	0.11
Combination therapy	44 (54.32%)	25 (44.64%)		16 (47.06%)	7 (26.92%)	
Rad-score (P25–P75)						
Target lesions	−0.46 (−0.60, −0.30)	−0.41(−0.55, −0.21)	0.27	−0.42 (−0.57, −0.21)	−0.39 (−0.57, −0.22)	0.54
Largest target lesion	−0.20 (−0.21, −0.18)	−0.19 (−0.20, −0.17)	0.10	−0.20 (−0.21, −0.16)	−0.19 (−0.20, −0.17)	0.89

^*^P value < 0.05.

For the sub-cohort analysis of patients who have both baseline and follow up CT scans at TP1 (n = 161), the two sets had identical distributions of DCR and clinical characteristics (**Table S2**). Among these patients, responders had lower percentage of distant metastasis compared to non-responders, with significant difference in training set (*P* = 0.02). There was no significant difference in other factors, including age, sex, smoking history, pathological type, and treatment strategy (**Table 2**).

**Table 2:** Characteristics of patients in Delta-radiomics analysis.

Characteristics	Training set		*P* value	Test set		*P* value
	Responders	Non-responders		Responders	Non-responders	
Age, median (P25–P75)	63 (35–84)	63 (44–78)	0.61	61 (29–75)	62 (36–77)	0.99	
Male	64 (35–84)	64 (44–78)		61 (29–75)	62 (36–70)		
Female	55 (43–79)	63 (59–74)		54 (48–64)	59 (37–77)		
Sex, No. (%)							
Male	60 (86.96%)	38 (88.37%)	0.83	24 (80.00%)	12 (63.16%)	0.19	
Female	9 (13.04%)	5 (11.63%)		6 (20.00%)	7 (36.84%)		
Smoking history, No. (%)							
Non-smokers	18 (26.09%)	13 (30.23%)	0.58	8 (26.67%)	6 (66.67%)	0.78	
Smokers	51 (73.91%)	30 (69.77%)		22 (73.33%)	13 (44.83%)		
Pathological type, No. (%)							
Adenocarcinoma	35 (50.72%)	26 (60.47%)	0.31	14 (46.67%)	9 (47.37%)	0.96	
Others	34 (49.28%)	17 (39.53%)		16 (53.33%)	10 (52.63%)		
Distant metastasis, No. (%)							
Absence	17 (24.64%)	3 (6.98%)	0.02^*^	8 (26.67%)	3 (15.79%)	0.06	
Presence	52 (75.36%)	40 (93.02%)		22 (73.33%)	16 (84.21%)		
Treatment strategy, No. (%)							
Monotherapy	32 (46.38%)	27 (62.79%)	0.09	17 (56.67%)	11 (57.89%)	0.93	
Combination therapy	37 (53.62%)	16 (37.21%)		13 (43.33%)	8 (42.11%)		
Rad-score of TP1 (P25–P75)	−0.47 (−0.75, −0.34)	−0.30 (−0.45, −0.18)	<0.01^*^	−0.44 (−0.76, −0.32)	−0.34 (−0.48, −0.19)	0.05	
Rad-score of Delta-RFs (P25–P75)							
Target lesions	−1.02 (−1.42, −0.57)	0.04 (−0.53, 0.54)	<0.01^*^	−0.97 (−1.63, −0.65)	−0.13 (−0.65, 0.12)	<0.01^*^	
Largest target lesion	−0.87 (−1.08, −0.51)	−0.20 (−0.62, 0.30)	<0.01^*^	−0.86 (−1.17, −0.63)	−0.46 (−0.59, −0.22)	0.03^*^	

^*^P value < 0.05.

### Feature Selection and Radiomics Nomogram Building Using Baseline Information

From the LL approach, three optimal features with respective nonzero coefficients in the training set were chosen to construct the radiomics signature prediction model (**Supplementary Equation 1**). The median Radscore of non-responders was slightly higher than responders in both training and test sets, but did not reach significant difference (*P* = 0.10, AUC = 0.59; *P* = 0.89, AUC = 0.51). From TL approach, seven features were chosen in the Radscore calculation formula (**Supplementary Equation 2**). Comparison of Radscore demonstrated no significance difference between the two response groups (*P* = 0.27, AUC = 0.56; *P* = 0.54, AUC = 0.53).

Combined nomograms that incorporated radiomics signature and clinical factor of distant metastasis were established. The ROC analysis exhibited fair prediction value of the developed model with an AUC of 0.65 (95% CI, 0.56 to 0.74) for LL approach and AUC of 0.64 (95% CI, 0.54 to 0.73) for TL approach in training set. The models carried out poorly in test sets (AUC = 0.52, 95% CI, 0.37 to 0.67; AUC = 0.61, 95% CI, 0.47 to 0.75).

### Delta-Radiomics Nomogram Building and Evaluation

**Figure 3:**
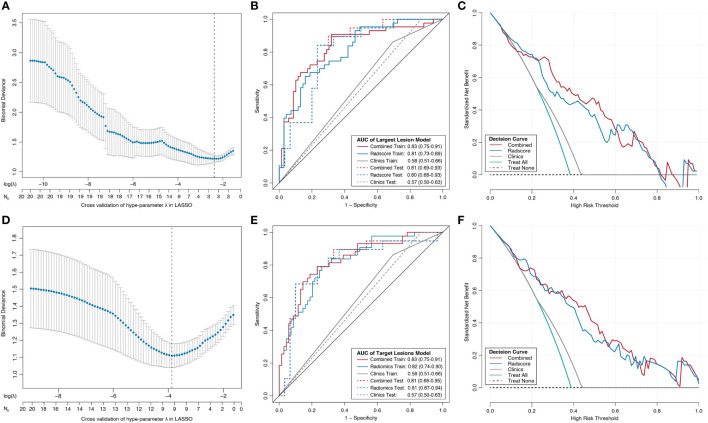
Radiomics feature selection using the least absolute shrinkage and selection operator (LASSO) binary logistic regression model, the developed nomograms with corresponding decision curves. **(A, D)** Tuning penalty factor (λ) in the LASSO model used 10-fold cross-validation *via* minimum criteria. The binomial deviance metrics (the y-axis) were plotted against log (λ) (the upper x-axis) and the number of selected features (the bottom x-axis). Blue dots indicate the average AUC for each model at the given λ, and vertical bars through the red dots show the upper and lower values of the binomial deviance in the cross-validation process. Dotted vertical black lines define the optimal λ, where the model provides its best fit to the data with optimal subset of variables. Receiver operating characteristic (ROC) curves comparison among combined radiomics model (red), radiomics model (blue), and clinical model (gray) for training set (solid line) and test set (dashed line) from the LL approach **(B)** and TL approach **(E)**. The combined radiomics model incorporating radiomics signature and clinical factor of distant metastasis showed the highest AUC. Decision curve analysis for the combined radiomics nomogram (red), radiomics signature (blue), and clinical model (gray) from the LL **(C)** approach and TL approach **(F)**. The y-axis indicates the net benefit; x-axis indicates threshold probability. The green line represents the assumption that all patients were responders. The black dotted line represents the hypothesis that no patients were responders.

Through the LASSO logistic regression analysis, three Delta-RFs were selected for LL approach (**Figure 3A**, **Supplementary Equation 3**). The Delta-Radscore was significantly higher in non-responders than in responders in both training (*P <*0.01) and test sets (*P* = 0.03) (**Figure S1A**). Responders presented lower level of Radscore at TP1 (*P <*0.01), and the difference was borderline significant in test set (*P* = 0.05) (**Figure S2A**). The developed Delta-radiomics signature showed a favorable result in predicting response status that produced an AUC of 0.81 in training set (95% CI, 0.73–0.89) and 0.80 in test set (95% CI, 0.68–0.93), respectively (**Figure 3B**). Specifically, this Delta-radiomics signature performed better prediction performance than radiomics signature constructed with radiomics features at TP1 (**Supplementary Equation 4**) (**Figure S2B**); however, the improvement did not showed significance in the Delong Test (*P* = 0.09, *P* = 0.16, respectively).

The Delta-radiomics signature for TL approach was developed using nine Delta-RFs (**Figure 3D**, **Supplementary Equation 5**). There was a significant difference in Delta-Radscore between responders and non-responders in training set (*P <*0.01), which was then confirmed in test set (*P <*0.01) (**Figure S1B**). In the ROC analysis, the Delta-radiomics signature prediction model yielded an AUC of 0.82 (95% CI, 0.74–0.90) in training set and 0.81 (95% CI, 0.67–0.94) in test set (**Figure 3E**).

A combined Delta-radiomics nomogram incorporating the developed delta-radiomics signature with clinical factor of distant metastasis was chosen as the best response status classifier (**Figures 2C, D**). The usefulness of combined Delta-radiomics nomogram for LL approach was confirmed in the ROC analysis with an AUC of 0.83 (95% CI, 0.75–0.91) for training set and an AUC of 0.81 (95% CI, 0.69–0.93) for test set (**Table 3**, **Figure 3B**). Meanwhile, combined Delta-radiomics nomogram for TL approach yielded an AUC of 0.83 (95% CI, 0.75–0.91) in training set and 0.81 (95% CI, 0.68–0.95) in test set (**Table 3**, **Figure 3E**), which was comparable with that from the LL approach (*P* = 0.92, *P* = 0.97). The prediction accuracy was 0.77 for the former model and 0.78 for the latter one (**Table S3**) without any significance (*P* = 1.00). The calibration curves of the combined Delta-radiomics nomograms showed good agreements between the nomogram prediction and actual observation (**Figures S1C, D**). The DCA (**Figures 3C, F**) indicated that when the threshold probability for a patient is within a range from 0 to 0.84, the combined Delta-radiomics nomograms add more net benefit than the “treat all” or “treat none” strategies from either the LL or TL approach.

**Table 3:** Multivariable logistic regression analyses.

Intercept and variable	Model 1 (target lesions)			Model 2 (largest target lesion)		
	Coefficient	Odds ratio (95% CI)	*P* value	Coefficient	Odds ratio (95% CI)	*P* value
Intercept	−0.48		0.36	−0.32		0.57
Delta Radiomics signature	1.50	4.47 (2.33, 9.59)	<0.01^*^	2.41	11.11 (4.03, 30.63)	<0.01^*^
Distant metastasis	0.94	2.56 (0.83, 7.89)	0.10^*^	1.27	3.55 (1.07, 11.75)	0.04^*^
C-index						
Training set	0.83 (0.75, 0.91)			0.83 (0.75, 0.91)		
Test set	0.81 (0.68, 0.95)			0.81 (0.69, 0.93)		

^*^P value < 0.05.

To control confounding factors, stratified analysis for treatment strategy was made (**Table S4**). There was no significant difference regarding DCR, pathological type, or distant metastasis between patients received monotherapy and those with combination therapy (*P* = 0.14, *P* = 0.90, *P* = 0.13). The Radscore and combined Delta-radiomics nomogram of monotherapy group demonstrated comparable performance to combination therapy group either from LL approach or from TL approach (all *P >*0.05 for AUCs comparison).

### Stratified Pretreatment PD-L1 Expression as a Predictor of Response Status

In the sub-cohort of 161 patients with available Delta-RFs, PD-L1 expression status was known for 66 patients. It was negative in 10 of 66 patients (15.15%), with an accuracy of 39.39% (26 of 66) in predicting 6-month response status. Significant superiority on accuracy (*P <*0.01) of radiomics-based models (up to 94.95%, **Table S5**) over pretreatment PD-L1 expression status was observed.

## Discussion

At present, radiological evaluation forms the objective basis of treatment response assessment criteria for lung cancer patients. The approach involves manually measuring changes in size of target lesions between baseline and follow-up CT scans in conjunction with RECIST guidelines (33, 37). Unfortunately, pure morphological criteria, even with modifications and refinements (i.e., iRECIST), are not sufficient because they only provide a consistent standard for management of data collected in clinical trials rather than clinical practice or therapy decisions (35, 38–41). Owing to its distinctive biologic mechanisms of action, immunotherapy can generate a tumor response pattern different from those found with cytotoxic chemotherapy or radiation therapy (42). Unconventional response patterns such as pseudoprogression and hyperprogression pose a major challenge to treating physicians, who aim to avoid either premature discontinuing the therapy too early in the treatment course or prolonging ineffective treatment that could put patients at higher risk of immune-related toxicity (43, 44). In this multicenter study, we did analysis on standard medical images that routinely used for monitoring therapeutic response to ICIs in advanced NSCLC patients from a radiomics-based approach. As demonstrated in this work, delta-radiomics based nomograms were developed as predictive biomarkers to identify patients who could derive the greatest therapeutic benefit from ICIs, which were successfully validated in an independent test set.

Considering of developing a cost-effective decision-support tool, we first construct a single-time-point radiomics signature from baseline CT scans to help stratifying patients to receive the most appropriate therapy strategy. In the context of lung cancer, radiomics studies typically extract features from the primary lung tumor, largest lung lesion, or one of the target lesions (19, 22, 23). By contrast, in this work, target lesions (up to five lesions per patient and up to two lesions per organ) were all included in the analysis. To the best of our knowledge, no previous studies have explored the capability of RFs of CT images for all target lesions in immunotherapy response evaluation. We suspect that this novel approach, which was more consistent with what we did in clinical practice regarding response evaluation of immune-based therapeutics, could reflect total tumor burden to some extent. In addition, we noticed that a few patients present both responding and progressive lesions (i.e. mix-response) at follow up examination. Under this circumstance, potential selection bias could be avoided in use of purposed TL approach comparing to LL approach.

The results demonstrated that nomograms incorporating baseline radiomics signature and clinical factor of distant metastasis did not exhibit high predictive value, which were inconsistent with prior studies (19, 21). We believe that such a discrepancy can be explained in part by the fact that RFs were extracted from plain CT imaging data rather than contrast enhanced CT images. Another possible cause is that patients receiving anti-PD 1 monotherapy and immunotherapy-based combinations were all included in the dataset, leading to the heterogeneous composition of our cohort. As combination of immunotherapy and chemotherapy regimen is now recommended as first-line therapy options for certain NSCLC patients according to NCCN recommendations (6), this study design is more in line with actual clinical situation. Moreover, the result of stratified analysis for treatment strategy confirmed that there was no significant difference in model efficacy between different treatment groups.

Although single time medical images especially those obtained at baseline are conventionally used for prediction, they do not contain information regarding treatment response. Delta-radiomics could offer abundant temporal-dependent information regarding therapy induced changes during the course of treatment (31, 45), and is relatively free of interference by factors that affect the reproducibility of quantitative image analysis. We proposed Delta-radiomics signature and compared it with single-time-point radiomics signature at TP1. Interestingly, Delta-radiomics signature of LL approach showed higher AUC, which agrees with a recent paper (26). Although we did not find significant difference of AUC between them, the lower 95% confidence interval of AUC at TP1 is 0.51 in the test set, indicating an insufficient diagnosis efficiency. Furthermore, Radscore between the two response groups had borderline significance with *P* value of 0.05 in the test set at TP1, suggesting that the radiomics signature might be slightly over-fitted to training set. Therefore, we can reasonably infer that Delta-radiomics could provide better predictive decision support. Meanwhile, we noticed that a decrease in sum of measures of target lesions did not guarantee benefit from immunotherapy. In this study, a transient tumor increase in size was encountered at TP1 in 15 patients, which was followed by a delayed response or stability and categorized as responders at 6 months of immunotherapy initiated. Hence, conventional CT interpretation, which relies on primarily sum of the target lesions, could not be a sensitive index for response assessment.Notably, the combined radiomics nomogram of LL approach achieved favorable predicting capacity. A combination of non-specific morphological information (i.e. major and least axis length) and contextual metrices of voxel intensity which depicted the diversity of convergent CT-value clusters probably reflecting agglomerate tissue areas (cancer cell nests or inflammation-induced necrosis) were included from both LL and TL approaches, so that a comprehensive representation of tumor evolutionary dynamics in the course of immunotherapy was promised.

This study is unique in that we conducted radiomics analysis in both lesion and patient level with a comparable performance. This observation highlights the feasibility and effectiveness of the utility of Delta-radiomics analysis on all target lesions, which could provide a consistent framework to iRECIST and overcome those confusions caused by mixed response pattern of immune-based therapeutics in NSCLC patients. More interestingly, our results showed that Delta-radiomics models outperformed pretreatment PD-L1 expression status in predicting response to ICIs in a subset of patients, and the combined model of TL approach had the highest accuracy. So far, the effectiveness of imaging-driven biomarkers with pretreatment CT images for prediction of PD-L1 expression in advanced NSCLC has been tentatively confirmed in several retrospective populations (46, 47), which enables investigators to validate the combination of PD-L1 expression signature with Delta-radiomics model for a better patient stratification and management in further prospective trials.

Our study has some limitations, the first of which is the heterogeneity of the cohorts, which could affect feature extraction and the procedure of analysis, even if several efforts has been made to weaken multicenter effect. Second, the sample size of the cohort was relatively small. Third, brain metastatic lesions were not chosen as target lesions in our analysis because multimodality approach is beyond the scope of this study. Given that the presence of distant metastasis is incorporated into the nomogram model as a clinical factor, the exclusion of brain metastatic lesion would not affect final prediction. Fourth, the potential biological underpinnings of radiomic features were not discussed in the current study, since relevant data that capturing tumor micro-environment was not available for this retrospective cohort. Finally, we had a limited follow-up period for some patients, and PFS and OS analyses were not done on this dataset. However, because of advanced tumor stage, our follow-up interval was deemed sufficient to provide clinically relevant information.

The results from our pilot study have shown that CT based Delta-radiomics biomarkers may facilitate treatment response prediction for NSCLC patients receiving immunotherapy with PD-1 ICIs. This procedure could be integrated into the normal clinical workflow without any additional cost.

## Data Availability Statement

The raw data supporting the conclusions of this article will be made available by the authors, without undue reservation.

## Ethics Statement

The studies involving human participants were reviewed and approved by Tianjin Medical University Cancer Institute and Hospital. Written informed consent for participation was not required for this study in accordance with the national legislation and the institutional requirements.

## Author Contributions

Conception and design: YiL, MW, YZ, and ZY. Literature research: YiL, MW, and YZ. Collection and assembly of data: all authors. Clinical studies: YiL, MW, and YZ. Data analysis and interpretation: YZ, SW, and YG. Manuscript writing: YiL, MW, YZ, and ZY. All authors contributed to the article and approved the submitted version.

## Funding

This work was supported by the National Natural Science Foundation of China (No. 81974277) and Demonstrative Research Platform of Clinical Evaluation Technology for New Anticancer Drugs (No. 2018ZX09201015).

## Conflict of Interest

The authors declare that the research was conducted in the absence of any commercial or financial relationships that could be construed as a potential conflict of interest.

The handling editor declared a past co-authorship with one of the author ZY.
