# Supplementary material for: Imaging Biomarkers to Predict and Evaluate the Effectiveness of Immunotherapy in Advanced Non-Small-Cell Lung Cancer
Source: Front Oncol. 2021 Mar 19;11:657615. doi: 10.3389/fonc.2021.657615 (PMC8017283; doi:10.3389/fonc.2021.657615)
Supplement: Supplementary file 1 [file DataSheet_1.docx]

Supplementary Material

# Supplementary Data

**CT scanning protocol**

1. 120 kVp with tube current adjusted automatically
2. Reconstruction thickness:

- 1.25mm for Discovery CT750 HD and Discovery CT750 scanner
- 1.5 mm for Siemens SOMATOM Definition AS+ scanner
- 4 mm for Canon Aquilion ONE scanner
- 5 mm for the other five scanners

1. Field of view (FOV): 36.5 cm to 44 cm
2. Matrix: 512×512
3. Reconstruction Kernel: Standard Algorithm (Std for GE, B30f for Siemens, or other equivalent)

Note.—Voxels were rescaled into 1.5mm isotropic since 1.5mm is both a median of double transverse pixel size and a median of slice thickness in our cohort which balanced the in-plane spatial resolution and volumetric interpolation between slices.

**Supplementary Equations**

Supplementary Equation 1:

Radscore = 0.003 × RLM_RunLengthNonuniformity_AllDirection_offset7_SD－0.05 × GLCM_ClusterShade_angle135_offset4 + 0.046 × Hist_MeanDeviation －0.191

*(IBSI Consensus of Standardization: Strong & very strong: 3, Moderate: 0, Weak:0)*

Supplementary Equation 2:

Radscore = 0.071 × GLCM_GLCMEnergy_AllDirection_offset1_SD－0.035 × GLCM_ClusterProminence_AllDirection_offset1_SD － 1.003 × GLCM_InverseDifferenceMoment_AllDirection_offset4＋0.528 × GLCM_InverseDifferenceMoment_angle135_offset7＋1.965 × GLCM_InverseDifferenceMoment_angle45_offset7－2.355 × RLM_ShortRunEmphasis_angle45_offset7＋3.789 × RLM_ShortRunEmphasis_angle90_offset7 －0.371

*(IBSI Consensus of Standardization: Strong & very strong: 7, Moderate: 0, Weak:0)*

Supplementary Equation 3:

Radscore = 0.256 × RLM_GreyLevelNonuniformity_angle90_offset1 ＋ 0.256 × LeastAxisLength ＋ 0.24 × GLSZM_ZonePercentage － 0.498

*(IBSI Consensus of Standardization: Strong & very strong: 2, Moderate: 1, Weak:0)*

Supplementary Equation 4：

Radscore = 0.141 × Flatness－0.153 × GLCM_Inertia_AllDirection_offset1_SD－0.032 × Hist_skewness－0.061 × SurfaceVolumeRatio－0.145 × GLCM_Inertia_AllDirection_offset7_SD －0.488

*(IBSI Consensus of Standardization: Strong & very strong: 3, Moderate: 0, Weak:0, Unknown 2)*

Supplementary Equation 5：

Radscore=0.348×Hist_Percentile90－0.181 × GLCM_GLCMEnergy_AllDirection_offset4_SD + 0.122 × GLCM_InverseDifferenceMoment_angle45_offset7－0.253 × Haralick_inverseDifferenceMoment－0.104 × RLM_LongRunEmphasis_angle135_offset4－0.337 × RLM_LongRunLowGreyLevelEmphasis_angle90_offset1 + 0.661 × GLSZM_ZonePercentage + 0.429 × LeastAxisLength + 0.082 × MajorAxisLength －0.53

*(IBSI Consensus of Standardization: Strong & very strong: 9, Moderate: 0, Weak:0)*

Note.—IBSI Consensus of Standardization was evaluated in digital phantom dataset.

# Supplementary Figures and Tables

## Supplementary Figures


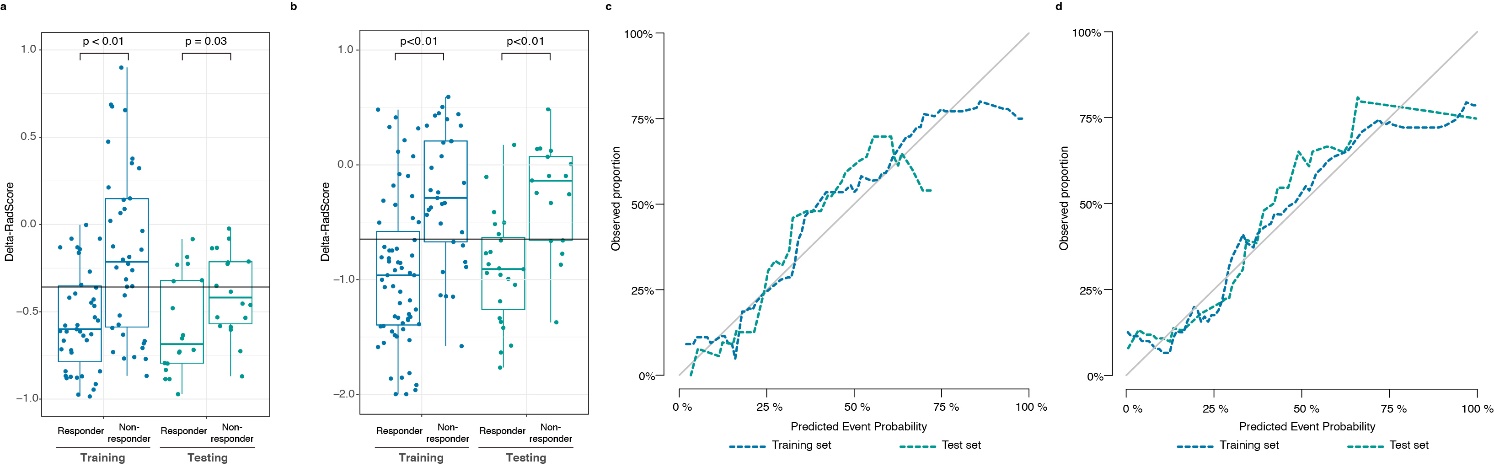


**Supplementary Figure 1.** Box and whisker plots depict Delta-Radscore comparison between responders and non-responders from LL approach **(a)** and TL approach **(b)**. Non-responders tend to have significantly higher Delta-Radscore in both sets (all *P<0.05*). Calibration curve analysis for the combined prediction Delta-radiomics nomogram in training cohort and validation cohort from LL approach **(c)** and TL approach **(d)**. The y-axis represents the actual outcome of non-responder rate. The x-axis represents the predicted probability of non-responder. The diagonal gray line represents an ideal prediction. The dashed line represents the performance of the Delta-radiomics nomogram in training set (in blue) and test set (in green) respectively, of which a closer fit to the diagonal black line represents a better prediction.

**
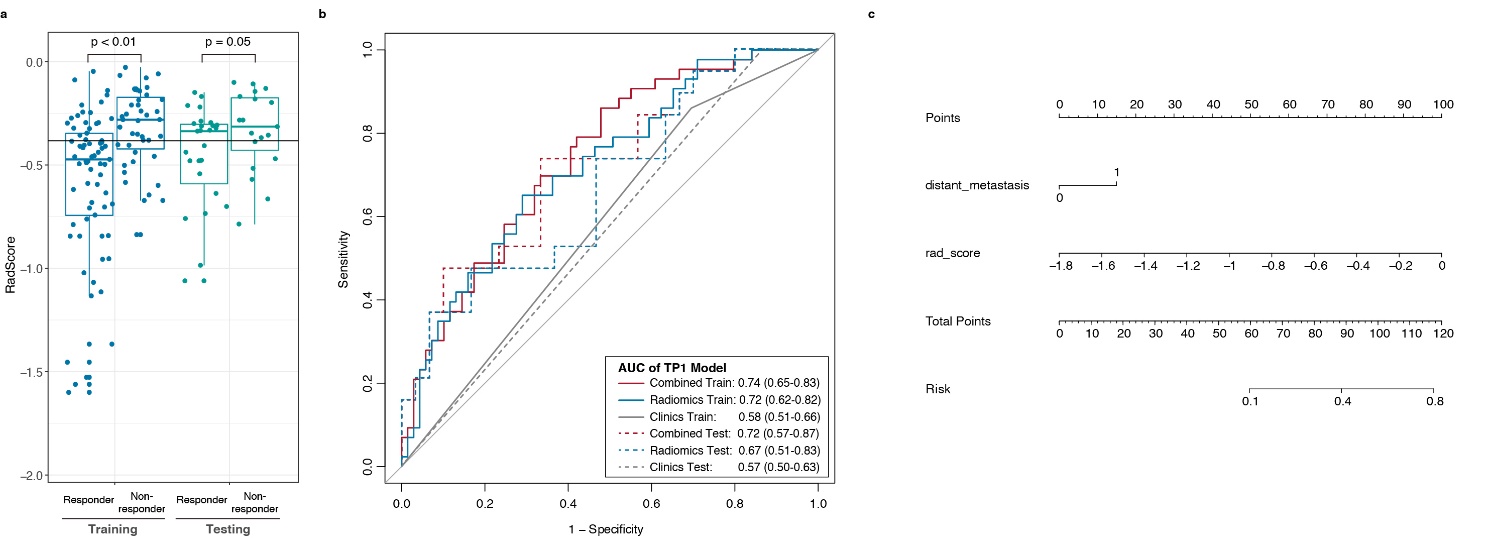
**

**Supplementary Figure 2.** **(a)** Box and whisker plots depict Radscore comparison between responders and non-responders at TP1. **(b)** ROC curves of radiomics signatures in training and test sets at TP1. **(c)** Nomogram of LL approach which were developed in training set.

## Supplementary Tables

**Table S1.** 402 radiomics features extracted using in-house software (Analysis Kit, version 3.2.5, GE Healthcare)

| **Feature Category** | **Number of features** | **Feature Name** |
| --- | --- | --- |
| First Order Histogram Features | 42 | 1. Min Intensity  2. Max Intensity  3. Median Intensity  4. Mean Value  5. Std Deviation  6. Mean Deviation  7. Relative Deviation  8. Variance  9. Range  10. Volume count  11. Voxel value  12. RMS  13. Skewness  14. Kurtosis  15. Uniformity  16. Histogram Energy  17. Histogram Entropy  18. Frequency Size  19. Percentile x (x=from 5 to 95, step=5)  20. Quantile y (y=0.025, 0.25, 0.5, 0.75, 0.975) |
| Haralick Features | 10 | 1. HaraEntroy  2. AngularSecondMoment  3. Contrast  4. HaraVariance  5. SumAverage  6. SumVariance  7. SumEntropy  8. DifferenceVariance  9. DifferenceEntropy  10. InverseDifferenceMoment |
| Gray Level Co-occurrence Matrix | 144 | 1. GLCMEnergy  2. GLCMEntropy  3. Inertia  4. Correlation  5. InverseDifferenceMoment  6. ClusterShade  7. ClusterProminence  8. HaralickCorrelation  Feature Angle=All, 0, 45, 90, 135, All_SD;  Offset=1, 4, 7 |
| Gray Level Run-length Matrix | 180 | 1. ShortRunEmphasis  2. LongRunEmphasis  3. GreyLevelNonuniformity  4. RunLengthNonuniformity  5. LowGreyLevelRunEmphasis  6. HighGreyLevelRunEmphasis  7. ShortRunLowGreyLevelEmphasis  8. ShortRunHighGreyLevelEmphasis  9. LongRunLowGreyLevelEmphasis  10. LongRunHighGreyLevelEmphasis  Feature Angle=All, 0, 45, 90, 135, All_SD;  Offset=1, 4, 7 |
| Gray Level Size-zone Matrix | 11 | 1. Small Area Emphasis  2. Large Area Emphasis  3. Intensity Variability  4. Zone-Size Variability  5. Zone Percentage  6. LowIntensitySmallAreaEmphasis  7. LowIntensityLargeAreaEmphasis  8. HighIntensitySmallAreaEmphasis  9. HighIntensityLargeAreaEmphasis  10. HighIntensityEmphasis  11. LowIntensityEmphasis |
| Morphological Features | 15 | 1. Sphericity  2. Surface Area  3. Surface Volume Ratio  4. Maximum 3D Diameter  5. Compactness1  6. Compactness2  7. Spherical Disproportion  8. Elongation  9. Flatness  10. LeastAxisLength  11. MajorAxisLength  12. MeshVolume  13. MinorAxisLength  14. OneVoxelVolume  15. VoxelVolume |

**Table S2.** Clinical Characteristics comparison between training set and test set

| **Variable** | **N=197 (entire cohort)** | | |  | **N=161 (sub-cohort for delta radiomics)** | | |
| --- | --- | --- | --- | --- | --- | --- | --- |
|  | **Training set** | **Test set** | ***P* value** |  | **Training set** | **Test set** | ***P* value** |
| Age, madian (P25-P75) | 64.0 (55.0-69.0) | 62.5 (53.0-67.0) | 0.09 |  | 63.5 (56.0-68.0) | 62.0 (55.0-70.0) | 0.15 |
| Sex, No. (%) |  |  |  |  |  |  |  |
| Male | 112(81.75%) | 53 (88.33%) | 0.60 |  | 98 (87.50%) | 36 (73.47%) | 0.92 |
| Female | 25(18.25%) | 7 (11.67%) |  |  | 14 (12.50%) | 13 (26.53%) |  |
| Smoking history, No. (%) |  |  |  |  |  |  |  |
| Non-smokers | 39 (28.47%) | 16 (26.67%) | 0.93 |  | 31 (27.68%) | 14 (28.57%) | 0.91 |
| Smokers | 98 (71.53%) | 44 (73.33%) |  |  | 81 (72.32%) | 35 (71.43%) |  |
| Pathological type, No. (%) |  |  |  |  |  |  |  |
| Adenocarcinoma | 66 (48.18%) | 31 (51.67%) | 0.65 |  | 61 (54.46%) | 23 (46.94%) | 0.12 |
| Others | 71 (51.82%) | 29 (48.33%) |  |  | 51 (45.54%) | 26 (53.06%) |  |
| Distant metastasis, No. (%) |  |  |  |  |  |  |  |
| Absence | 26 (19.00%) | 10 (16.67%) | 0.99 |  | 20 (17.86%) | 11 (22.45%) | 0.81 |
| Presence | 111 (81.00%) | 50 (83.33%) |  |  | 92 (82.14%) | 38 (77.55%) |  |
| Treatment strategy, No. (%) |  |  |  |  |  |  |  |
| Monotherapy | 68 (49.64%) | 37 (61.67%) | 0.22 |  | 59 (52.68%) | 28 (57.14%) | 0.39 |
| Combination therapy | 69 (50.36%) | 23 (38.33%) |  |  | 53 (47.32%) | 21 (42.86%) |  |
| Disease control rate (%) | 59.12 | 56.67 | 0.75 |  | 61.61 | 61.22 | 0.96 |

**Table S3.** ROC analysis for delta-radiomics nomograms

| **Variables** | **Model 1 (Largest Lesion Approach)** | | **Model 2 (Target Lesions Approach)** | |
| --- | --- | --- | --- | --- |
|  | **Training set (n = 112)** | **Test set (n = 49)** | **Training set (n = 112)** | **Test set (n = 49)** |
| Accuracy | 0.77 (95% CI: 0.68–0.84) | 0.80 (95% CI: 0.66–0.90) | 0.78 (95% CI: 0.69–0.85) | 0.82 (95% CI: 0.68–0.91) |
| Sensitivity | 0.65 | 0.68 | 0.70 | 0.84 |
| Specificity | 0.91 | 0.87 | 0.84 | 0.80 |

**Table S4.** Stratified analysis of different treatment strategy

| **Variables** | Monotherapy (n=87) | | | |  | Combination therapy (n=74) | | | |
| --- | --- | --- | --- | --- | --- | --- | --- | --- | --- |
|  | Largest target lesion approach | | Target lesions approach | |  | Largest target lesion approach | | Target lesions approach | |
|  | Rad-score | Combined model | Rad-score | Combined model |  | Rad-score | Combined model | Rad-score | Combined model |
| AUC | 0.79 | 0.81 | 0.83 | 0.84 |  | 0.82 | 0.83 | 0.77 | 0.79 |
| Specificity | 0.71 | 0.67 | 0.86 | 0.88 |  | 0.60 | 0.70 | 0.70 | 0.76 |
| Sensitivity | 0.76 | 0.92 | 0.74 | 0.74 |  | 0.96 | 0.88 | 0.83 | 0.79 |
| Accuracy | 0.74 | 0.78 | 0.80 | 0.82 |  | 0.72 | 0.76 | 0.74 | 0.77 |

**Table S5.** Performance comparison of models incorporating Delta-radiomics biomarkers against that of PD-L1 expression status in predicting response to ICIs.

| Variables | Largest target lesion approach | | Target lesions approach | |
| --- | --- | --- | --- | --- |
|  | Rad-score | Combined model | Rad-score | Combined model |
| Accuracy | 80.30% | 80.30% | 81.82% | 95.45% |
| $\chi^{2}$ | 22.846 | 22.846 | 24.717 | 28.758 |
| *P*-value | < 0.001 | < 0.001 | < 0.001 | < 0.001 |
